# Incidence and Predictors of Immune Reconstitution Inflammatory Syndrome in a Rural Area of Mozambique

**DOI:** 10.1371/journal.pone.0016946

**Published:** 2011-02-28

**Authors:** Emilio Letang, José M. Miró, Tacilta Nhampossa, Edgar Ayala, Joaquim Gascon, Clara Menéndez, Pedro L. Alonso, Denise Naniche

**Affiliations:** 1 Centro de Investigação em Saúde de Manhiça (CISM), Manhiça, Maputo, Mozambique; 2 Infectious Diseases Service, Hospital Clínic/IDIBAPS, Universitat de Barcelona, Barcelona, Spain; 3 Barcelona Centre for International Health Research (CRESIB), Hospital Clínic/IDIBAPS, Universitat de Barcelona, Barcelona, Spain; Institute of Infectious Diseases and Molecular Medicine, South Africa

## Abstract

**Background:**

There is limited data on the epidemiology of Immune Reconstitution Inflammatory Syndrome (IRIS) in rural sub-Saharan Africa. A prospective observational cohort study was conducted to assess the incidence, clinical characteristics, outcome and predictors of IRIS in rural Mozambique.

**Methods:**

One hundred and thirty-six consecutive antiretroviral treatment (ART)-naïve HIV-1-infected patients initiating ART at the Manhiça district hospital were prospectively followed for development of IRIS over 16 months. Survival analysis by Cox regression was performed to identify pre-ART predictors of IRIS development.

**Results:**

Thirty-six patients developed IRIS [26.5%, incidence rate 3.1 cases/100 persons-month of ART (95% CI 2.2–4.3)]. Median time to IRIS onset was 62 days from ART initiation (IQR 35.5–93.5). Twenty-five cases (69.4%) were “unmasking”, 10 (27.8%) were “paradoxical”, and 1 (2.8%) developed a paradoxical worsening followed by the unmasking of another condition. Systemic OI (OI-IRIS) accounted for 47% (17/36) of IRIS cases, predominantly of KS (8 cases) and TB (6 cases) IRIS. Mucocutaneous IRIS manifestations (MC-IRIS) accounted for 53% (19/36) of IRIS events, mostly tinea (9 cases) and herpes simplex infection (3 cases). Multivariate analysis identified two independent predictors of IRIS development: pre-ART CD4 count <50 cells/µl (HR 2.3, 95% CI 1.19–4.44, p = 0.01) and body mass index (BMI) <18.5 (HR 2.15, 95% CI 1.07–4.3, p = 0.03). The pre-cART proportion of activated T-cells, as well as the immunologic and virologic response to ART were not associated with IRIS development. All patients continued on ART, 7 (19.4%) required hospitalization and there were 3 deaths (8.3%) attributable to IRIS.

**Conclusions:**

IRIS is common in patients initiating ART in rural Mozambique. Pre-ART CD4 counts and BMI can easily be assessed at ART initiation in rural sub-Saharan Africa to identify patients at high risk of IRIS, for whom close supervision is warranted.

## Introduction

Combined Antiretroviral therapy (cART) has led to dramatic declines in the morbidity and mortality secondary to HIV infection and AIDS-associated opportunistic illness (OI) [1], [2]. However, some patients initiating cART experience clinical deterioration, which has been called the immune reconstitution inflammatory syndrome (IRIS). IRIS is observed in patients who demonstrate a good response to ART but experience a paradoxical clinical worsening. In these patients, the rapid restoration of functionally active antigen-specific cells after ART is hypothesized to initially lead to an immunopathologic rather than protective effect, resulting in worsening of a known condition (“paradoxical” IRIS) or a presentation of previously unrecognized OI (“unmasking” IRIS) [3]. IRIS has been related to a variety of etiologies, including tuberculosis (TB), non-tuberculous mycobacteria, cryptococcus, herpesviruses, and Kaposi's Sarcoma (KS) [3], [4], [5], [6].

Previous studies, have reported an IRIS incidence of 8–32% of patients initiating ART [7], [8], [9], [10], [11]. A recent meta-analysis found IRIS incidence to be of 16.1% of unselected patients starting cART, varying according to the provoking pathogen [12]. Disparities may be explained by differences in OIs investigated, IRIS case definitions, and study populations. Remarkably, few studies have characterized IRIS in sub-Saharan Africa (SSA), where a high burden of IRIS related-OI exists. In a prospective study conducted in South Africa, 10.4% of patients initiating cART developed IRIS, mostly associated with TB, dermatological manifestations, varicella zoster infection, herpes simplex infection, and Cryptococcal meningitis [10]. More studies are needed to better characterize IRIS in rural SSA.

Four major risk factors for IRIS development have been identified: Low CD4 count at cART initiation [7], [8], [10], [11]; (ii) robustness of immunologic and virologic response to cART [9], [13]; (iii) high antigenic burden of an OI at cART initiation [14], and (iv) early initiation of cART after some OI, particularly TB [5], [9]. Substantial heterogeneity exists across studies, but the predictor most consistently associated with IRIS development is a low pre-cART CD4 count.

We conducted a prospective cohort study in a rural area of Mozambique aimed at assessing the incidence, clinical characteristics, outcome and predictors of IRIS in this area.

## Methods

### Study population and Ethics statement

This study was conducted from April 2006 through May 2008 at the Manhiça District Hospital (MDH), in southern Mozambique. All HIV-1 positive non-pregnant adults living in the study area, attending the HIV/AIDS voluntary counselling and testing services at the MDH, and meeting cART criteria were invited to participate. CART criteria and regimens used are specified elsewhere [15].

All patients gave written informed consent, and the study protocol was approved by the Mozambican National Bioethics Committee (ref. 44/CNBS/06) and the Hospital Clinic of Barcelona Ethics Review Committee (ref. CEIC/3158).

### Data collection

Visits were scheduled according to the Mozambican Guidelines and included a pre-treatment assessment and regular assessments at week 2 and months 4, 10 and 16 after cART initiation. Baseline serologic assessment of hepatitis B virus (HBV), hepatitis C virus (HCV), toxoplasma, syphilis, Human T-lymphotropic virus (HTLV), and human herpesvirus-8 (HHV-8) was conducted. Each scheduled visit included a clinical exam and monitoring of plasma HIV-1 RNA, CD4, CD8 counts and T-cell activation, WBC, RBC and platelet counts, and hepatic and renal function. Passive surveillance for OI was performed throughout the study to detect potential IRIS cases.

### HIV-1 RNA determinations

Plasma HIV-1 viral load was measured using a reverse transcriptase (RT) polymerase chain reaction (PCR) technique (Amplicor, Roche Monitor-v1.5) from cryopreserved samples. The lower limit of detection was 400 copies/ml.

### Immunology and haematological determinations

CD4 counting was performed after staining fresh whole blood samples with labelled antibodies: CD4, CD3, CD8, and CD45 in TruCount tubes (BD Biosciences, California, USA).

To access the percentage of activated CD4 and CD8 T-lymphocytes, cell staining were performed with CD4, CD38 and HLA-DR or with CD3, CD8, CD38 and HLA-DR respectively. Activated T-cells were defined as those CD8 or CD4 T cells expressing both CD38 and HLA-DR surface markers.

### Assessment of baseline co-infections

Determine (Abbott, Illinois, USA) was used for HBV surface antigen testing. Rapid Plasma Reagin was used for syphilis screening (HUMAN-diagnostics, Wiesbaden, Germany) and a treponemal ELISA-Trepanostika (BioMérieux, Netherlands) for confirmation. The remaining serologies were assessed by ELISA: HCV (HUMAN-diagnostics, Wiesbaden, Germany), HTLV I/II (Murex, Abbott, Illinois, USA), and HHV-8 (Biotrin, Dublin, Ireland).

TB cases included those patients smear positive for acid-fast bacilli or culture positive and those without microbiological confirmation but with compatible symptoms or signs [16].

### IRIS case definition

To define IRIS, we used the criteria proposed by French *et al* [5]. IRIS was defined as an abrupt clinical worsening of an existing condition (“paradoxical” IRIS) or new presentation of a previously unknown OI (“unmasking” IRIS) following cART initiation, and either with a concomitant reduction of at least 1 log_10_ of HIV-1 RNA levels at the time of IRIS or with 2 of the 3 minor criteria: (1) increased CD4 count after ART; (2) increase in an immune response specific to the relevant pathogen; (3) spontaneous resolution of disease.

Additionally, the exclusion of other causes of worsening was required: (i) expected clinical course of OI, given cART; (ii) drug toxicity; (iii) other infection or inflammatory condition; (iv) withdrawal of previously effective therapy; and (v) failure of ART: based on either non-adherence or on HIV RNA assessment [17].

Based on the clinical manifestation, IRIS cases were categorized as systemic OI-related IRIS (OI-IRIS) and mucocutaneous-IRIS (MC-IRIS).

Finally, IRIS cases were classified as “confirmed” or “probable” cases. “Confirmed” cases were those who fulfilled IRIS criteria at the time of the event. “Probable” IRIS cases were those who fulfilled criteria at the 4-month visit but had incomplete data at the time of the event, or those for whom competing diagnoses had not been adequately excluded.

To define all IRIS cases, the agreement of 2 clinicians (E.L and J.M.M) was required.

### Statistical analysis

Baseline and follow-up comparisons between patients who did and who did not develop IRIS were made using chi-square for categorical variables, and the Wilcoxon rank-sum test/Kruskal-Wallis for continuous variables.

The association between pre-cART variables and the time to develop IRIS was evaluated using survival analysis methodology. Differences between patients who did and who did not develop IRIS were tested in a univariate analysis using the Cox proportional hazards models to compute the hazard ratio (HR) of developing IRIS and corresponding 95% confidence interval (CI). Independent risk factors were identified in the multivariate analysis using a forward selection procedure, using univariate p<0.12 as inclusion criteria and the likelihood ratio test to assess the relative fit of the different models. A final model was derived after assessment for interaction between the relevant exposures. Kaplan-Meier survival estimates of IRIS development by the independent predictors identified were generated and the logrank test was used to compare the different survival curves.

All statistical analyses were performed using STATA 11 (STATA Corp., Texas, USA).

## Results

### Cohort patient characteristics

Between April and November 2006, 143 consecutive ART-naive HIV-1–positive adults with cART criteria were enrolled. Of these, 7 patients were lost to follow-up before any medical visit and were not included in the cohort analysis. Of the 136 patients included 4 (2.9%) were lost to follow-up between enrolment and 10 months.

Table 1 summarizes the pre-cART characteristics of the 136 patients included in the cohort analysis. All patients were of African ethnicity, predominantly female (58.8%), and with a median age at cART initiation of 36 years [Interquartile range (IQR) 29–45)]. The median baseline CD4 count was 115 cells/µl (IQR 32.5–183.5), and the median HIV viral load was 5.14 log_10_ copies/ml (IQR 4.8–5.4, Table 1).

**Table 1:** Summary of patient characteristics before cART initiation according to the development of IRIS.

	Total patients(n = 136)	IRIS patients(n = 36)	Non-IRIS patients(n = 100)	P value
**Age**, median (IQR)	36(29–45)	34.5(29–39.5)	37.5(29.5–45)	0.17
18–30 years, n(%)	41 (30.5)	13 (36.1)	28 (28)	
31–40 years, n(%)	47 (34.6)	15 (41.7)	32 (32)	
>40 years, n(%)	48 (35.3)	8 (22.2)	40 (40)	
**Gender**, n(%)				
Female	80 (58.8)	21 (58.3)	59 (59)	0.94
Male	56 (41.2)	15 (41.7)	41 (41)	
**African ethnicity**, n(%)	136 (100)	36 (100)	100 (100)	
**Body Mass index**				
Median (IQR)	21 (18-6–22.9)	20 (18–21.3)	21.3 (19–23)	0.009
<18.5, n(%)	32 (23.5)	12 (33.3)	20 (20)	0.1
**Karnofsky Performance Scale >80%, n(%)**	115 (84.6)	28 (77.8)	87 (87)	0.19
**HIV WHO stage** ^a^				
I–II	30 (22.6)	5 (13.9)	25 (25.8)	0.145
III–IV	103 (77.4)	31 (86.1)	72 (74.2)	
**CD4 lymphocyte counts**				
Median cells/µL (IQR)^b^	115(32.5–183.5)	68(20.5–156.5)	130.5(42.5–204.5)	0.059
>200 cells/µL, n(%)	31 (22.8)	5 (13.9)	26 (26)	
50–200 cells/µL, n(%)	62 (45.6)	14 (38.9)	48 (48)	
<50 cells/µL, n (%)^b^	43 (31.6)	17 (47.2)	26 (26)	
**CD8 lymphocyte counts**				
Median cells/µL (IQR)	551 (221.5–1115.5)	618 (193–834.5)	544.5 (221.5–1164)	0.69
**Percentage of activated CD4 cells**				
Median percentage (IQR)	33.4 (23.8–46.3)	32.9 (23.8–51.7)	33.4 (23.8–43.4)	0.88
**Percentage of activated CD8 cells**				
Median percentage (IQR)	66 (57.2–74.9)	65.8 (58.2–74.4)	66.3 (55.2–75.3)	0.64
**Percentage of naïve CD4 cells**				
Median percentage (IQR)	7.5 (2.4–19.3)	9.4 (3.4–20.7)	6.36 (2.4–18.4)	0.56
**Plasma HIV viral load**				
Median log_10_ copies/ml (IQR)	5.14(4.8–5.4)	5.17(4.96–5.4)	5.14(4.8–5.4)	0.73
>5 log_10_ copies/ml (IQR)	83 (61)	26 (72.2)	57 (57)	
**CART regimen**, n(%)				
d4T/3TC/NVP	85 (62.5)	24 (66.7)	61 (61)	0.38
d4T/3TC/EFV	22 (16.2)	5 (13.9)	17 (17)	
AZT/3TC/EFV	23 (16.9)	4 (11.1)	19 (19)	
AZT/3TC/NVP	6 (4.4)	3 (8.3)	3 (3)	

*Baseline WHO stage: 3 missing values; d4T: Stavudine; 3TC: Lamivudine; NVP: nevirapine; EFV: efavirenz.

Fourteen patients (10.3%) had a history of previous TB. Forty (29.4%) were on TB treatment at cART initiation, with a median time from TB treatment initiation to cART onset of 66.5 days (IQR 49.5–126). Other pre-cART diagnosis included HBV co-infection (18/136, 13.2%), syphilis (16/136, 11.8%), KS (13/136, 9.6%), HCV co-infection (8/136, 5.9%), and cerebral cryptococcosis (2/136, 1.5%).

All patients received cART, nevirapine-based for 91 patients (66.9%), and efavirenz-based for 45 patients (33.1%).

### Incidence and clinical description of IRIS cases

The 136 patients contributed 1151.7 person-month (p-m) of follow-up, with a median time of 9.8 months (IQR 2.3–16.1).

Thirty-six patients (26.5%) developed IRIS [incidence rate 3.1 cases/100 p-m of ART (95% CI 2.2–4.3)]. Of these, 30 (83.3%) were “confirmed”, and 6 (16.7%) were “probable” cases. The median time to IRIS development was 62 days (IQR 35.5–93.5) from cART initiation. Twenty-five cases (69.4%) were “unmasking”, 10 (27.8%) were “paradoxical”, and 1 (2.8%) developed a paradoxical worsening followed by the unmasking of another condition. Diagnosis included systemic OI (OI-IRIS) in 17 (47.3%) cases [KS (8/36, 22.2%), TB (6/36, 16.7%) including 2 patient with both KS-IRIS and TB-IRIS, interstitial pneumonitis of unknown etiology (2/36, 5.6%), *Pneumocystis jirovecii* pneumonia (1/36, 2.8%), cervical cancer (1/36, 2.8%), and *Strongyloides stercoralis* diarrhea (1/36, 2.8%)] and muco-cutaneous manifestations (MC-IRIS) in the remaining 19 patients (52.7%) [Tinea (9/36, 25%), herpes simplex infection (3/36, 8.3%), genital warts (2/36, 5.6%), varicella zoster infection (2/36, 5.6%), recurrent oral aphtosis ulcers (1/36, 2.8%), impetigo (1/36, 2.8%) and seborrheic dermatitis (1/36, 2.8%), Table 2].

**Table 2:** Clinical Characteristics of IRIS cases (n = 36).

	Time to IRIS (days)	Baseline CD4(cells/µL)	Baseline HIV RNA (log_10_ copies/ml)	IRIS CD4(cells/µL)	IRIS HIV RNA (log_10_ copies/ml)	Delta CD4(cells/µL)	Delta HIV RNA (log_10_ copies/ml)	Diagnosis	IRIS type	Outcome
1	105	136	4.52	184	2.30	48	2.22	Tinea corporis	P	R
2	36	107	5.27	156	2.30	49	2.97	Genital warts	P	R
3	78	66	4.94	163	2.30	97	2.64	Genital warts	P	R
4	36	133	5.11	506	2.30	373	2.81	KS	P	R
5	21	41	5.40	168	3.08	127	2.32	KS	P	R
6	41	538	5.03	669	2.30	131	2.73	KS	P	D (non-IR)
7*	10	233	5.53	*N/A*	*N/A*	*N/A*	*N/A*	KS	P	D (IR)
8	34	7	4.51	176	2.30	169	2.21	PCP	P	R
9	96	0	5.21	203	2.69	203	2.52	Strongyloides stercoralis diarrhea	P	R
10	39	13	5.35	333	2.30	320	3.05	TB lymphadenitis	P	R
11	64	40	5.25	144	2.30	104	2.95	Pulmonary TB/KS	P/U	R
12	58	236	5.43	247	2.30	11	3.13	Tinea cruris	U	R
13	24	43	4.99	162	2.30	119	2.69	Genital herpes	U	R
14*	126	6	5.06	15	*N/A*	9	*N/A*	Tinea corporis	U	R
15	11	455	5.35	558	2.88	103	2.47	Genital herpes	U	R
16	214	31	5.25	255	2.30	224	2.95	Cervical cancer	U	D (IR)
17	76	112	5.62	215	2.30	103	3.32	Tinea corporis	U	R
18	185	1	4.53	66	2.30	65	2.23	Genital herpes	U	R
19	8	165	4.36	199	2.96	34	1.40	Herpes zoster	U	R
20	82	1	4.63	377	2.30	376	2.33	Tinea faciei	U	R
21*	61	153	4.61	27	2.76	−126	1.85	Tinea faciei	U	D (non-IR)
22	87	6	5.43	92	2.30	86	3.13	Herpes Zoster	U	R
23*	159	182	4.48	363	2.93	181	1.55	Tinea faciei	U	R
24*	10	110	5.12	*N/A*	*N/A*	*N/A*	*N/A*	Inerstitial pneumonitis	U	D (IR)
25*	48	24	5.01	158	*N/A*	134	*N/A*	Tinea faciei	U	R
26	76	17	5.27	45	2.30	28	2.97	Pulmonary TB	U	R
27	161	71	5.55	77	2.30	6	3.25	KS	U	R
28	81	401	5.11	714	3.79	313	1.32	Tinea cruris	U	R
29	21	84	5.27	155	1.40	71	3.87	Impetigo	U	D (non-IR)
30	197	165	5.13	232	2.30	67	2.83	KS	U	R
31	39	34	5.34	521	2.30	487	3.04	Inerstitial pneumonitis	U	R
32	64	70	5.54	176	3.83	106	1.71	Recurrent oral aftae	U	R
33	126	160	4.76	474	2.30	314	2.46	Seborrhoeic dermatitis	U	R
34	91	2	5.03	89	2.30	87	2.73	TB lymphadenitis	U	R
35	35	24	5.64	21	2.93	−3	2.71	Milliary TB/KS	U	R
36	56	43	5.37	240	1.40	197	3.97	Pleuro-pulmonary TB	U	R
Median	63	68	5	180	2.30	104	3.2			

*Probable cases. The rest are confirmed cases; N/A: Not available; P: Paradoxical IRIS; U: Unmasking IRIS; R: Recovery; D: Death; IR: IRIS related death.

At the time of IRIS development, CD4 counts were available for 34/36 patients (94.4%) and HIV RNA for 32/36 (88.9%). Ninety-four percent (32/34) showed an increase in CD4 counts, and 32/32 showed >1 log_10_ decrease in HIV RNA compared to baseline. The median CD4 count was 180 cells/µl (IQR 144–333), representing a median increase of 104 cells/µl (IQR 49–197) from pre-cART levels in IRIS patients. The median HIV RNA was 1.12 log_10_ copies/ml (IQR 0.3–2.7), with a median decrease of 3.2 log_10_ copies/ml (IQR 1.9–4.7) from pre-cART levels (Table 2).

All patients continued on cART. Four (11.1%) received corticosteroids and 7 (19.4%) Non-Steroidal Anti-inflammatory drugs (NSAID). Seven IRIS cases (19.4%) required hospitalization and 6 died (16.67%), 3 of which were directly attributable to IRIS [KS-IRIS (patient 7), cervical cancer (patient 16) and interstitial pneumonitis of unknown etiology (patient 24)]. The other 3 deaths occurred with a median of 8 weeks (IQR 4–40) after the IRIS event, and were attributed to a gastroenteritis with severe dehydration (patient 6), cerebral toxoplasmosis (patient 21), and to unknown causes (patient 29). The mortality in the non-IRIS patients was 15% (15/100), similar to the mortality observed in IRIS patients (p = 0.81).

### Risk Factors Associated With IRIS Development

Table 3 shows the incidence rate of IRIS by the different baseline variables assessed and the univariate and multivariate analysis of IRIS predictors. Univariate analysis showed an association between IRIS development and the following pre-cART variables: Body Mass Index (BMI) <18.5, Karnofsky Scale <80%, CD4 counts <50 cells/µl, and haematocrit <30%.

**Table 3:** Univariate and multivariate analysis by Cox regression of pre-cART factors potentially contributing to IRIS development (n = 136).

	Category	n	IRIS, n (%)	p-m	IRIS rate per 100 p-m, rate (95% CI)	Univariate HR (95% CI)	P value	Multivariate HR (95% CI)	P value
**Age** (years)	18–30	41	13 31.7)	2.9	4.5 (2.6–7.8)	1			
	31–40	47	15 (31.2)	3.6	4.1 (2.5–6.8)	0.97 (0.46–2)	0.94		
	>40	48	8 (16.7)	5	1.6 (0.8–3.2)	0.45 (0.19–1.1)	0.079		
**Gender**	Male	56	15 (26.8)	4.3	3.5 (2.1–5.8)	1			
	Female	80	21(26.2)	7.2	2.9 (1.8–4.5)	0.93 (0.48–1.8)	0.83		
**BMI**	≥18.5	104	24 (23.1)	9.9	2.4 (1.6–3.6)	1	0.026	1	0.03
	<18.5	32	12 (37.5)	1.6	7.4 (4.2–13)	2.21 (1.1–4.4)		2.1 (1.1–4.3)	
**Karnofsky Scale**	≥80%	115	28 (24.3)	10.7	2.6 (1.8–3.8)	1	0.009		
	<80%	21	8 (38.1)	0.8	10 (5–20)	2.87 (1.3–6.3)			
**HIV WHO stage** a	I–II	30	5 (16.7)	3.1	1.6 (0.7–3.8)	1	0.11		
	III–IV	103	31 (30.1)	8.4	3.7 (2.6–5.3)	2.15 (0.8–5.5)			
**Previous OI**	No	70	17 (24.3)	6.2	2.7 (1.7–4.4)	1	0.45		
	Yes	66	19 (28.8)	5.3	3.5 (2.3–5.6)	1.28 (0.7–2.5)			
**CD4 counts** (cells/µL)	≥50	93	19 (20.4)	8.7	2.2 (1.4–3.4)	1	0.01	1	0.01
	<50	43	17 (39.5)	2.8	6.1 (3.8–9.9)	2.34 (1.21–4.5)		2.3 (1.2–4.4)	
**CD8 counts** (cells/µL)	<551*	68	16 (23.5)	5.8	2.8 (1.7–4.5)	1	0.6		
	≥551	68	20 (29.4)	5.8	3.5 (2.2–5.4)	1.19 (0.6–2.3)			
**HIV viral load** (log_10_ cp/ml)	<5	53	10 (18.9)	5	2 (1.1–3.7)	1	0.12		
	≥5	83	26 (31.3)	6.5	4 (2.7–5.9)	1.79 (0.9–3.7)			
**Activated CD4 cells** (%)	<33.4*	68	18 (26.5)	5.9	3 (1.9–4.8)	1	1		
	≥33.4	68	18 (26.5)	5.6	3.2 (2–5.1)	1 (0.5–1.9)			
**Activated CD8 cells** (%)	<66*	68	19 (27.9)	5.8	3.3 (2.1–5.1)	1	0.7		
	≥66	68	17 (25)	5.7	2.96 (1.8–4.8)	0.9 (0.47–1.7)			
**Naïve CD4 cells** (%)	<7.5	68	15 (22.1)	6	2.5 (1.5–4.1)	1			
	≥7.5	68	21 (30.9)	5.5	3.8 (2.5–5.9)	1.58 (0.81–3.1)	0.18		
**Total WBC counts** (×10^3^/µL)	≥4	92	23 (25)	7.6	3 (2–4.5)	1	0.53		
	<4	44	13 (29.5)	3.9	3.4 (1.9–5.8)	1.24 (0.63–2.4)			
**Haematocrit** (%)	≥30	98	21 (21.4)	9.1	2.3 (1.5–3.5)	1	0.016		
	<30	38	15 (39.5)	2.4	6.3 (3.8–10.4)	2.25 (1.16–4.4)			
**ALT level** (UI/l)	<55	114	28 (24.6)	9.7	2.9 (2–4.2)	1	0.3		
	<55	22	8 (36.4)	1.8	4.5 (2.3–9.1)	1.5 (0.69–3.3)			
**cART regimen**	NVP based	91	27 (29.7)	7.5	3.6 (2.5–5.2)	1	0.3		
	EFV based	45	9 (20)	4	2.2 (1.2–4.3)	0.67 (0.31–1.42)			
**CD4 – BMI** (risk category)	1 (high)	74	14 (18.9)	7.5	1.9 (1.1–3.2)	1	0.3		
	2	19	5 (26.3)	1.3	3.8 (1.6–9.2)	1.67 (0.6–4.63)	0.109		
	3	30	10 (33.3)	2.4	4.1 (2.2–7.6)	1.94 (0.86–4.37)	<0.001		
	4 (low)	13	7 (53.8)	0.3	21.9 (10.5–5)	5.38 (2.14–13.5)			

a Baseline WHO stage: 3 missing values; n: number of patients; p-m: persons-month of follow-up; HR: Hazard Ratio; CI: Confidence Interval; BMI: Body Mass Index; NVP: nevirapine; EFV: efavirenz; CD4 – BMI: combination of both independent predictors; Category 1: CD4 ≥50 cells/µL and BMI ≥18.5; Category 2: CD4 ≥50 cells/µL and BMI <18.5; Category 3: CD4 <50 cells/µL and BMI ≥18.5; Category 4: CD4 <50 cells/µL and BMI <18; * median.

Multivariate analysis identified two independent IRIS pre-cART predictors: CD4 <50 cells/µl (HR 2.3, 95% CI 1.19–4.44, p = 0.01) and BMI <18.5 (HR 2.15, 95% CI 1.07–4.3, p = 0.03). The combination of both predictors was associated with a greater than 5-fold hazard of IRIS development compared with the patients with a CD4 count ≥50 cells/µl and a BMI ≥18.5 (HR 5.38, 95% CI 2.14–13.54, p<0.001, Table 3).

**Figure 1:**
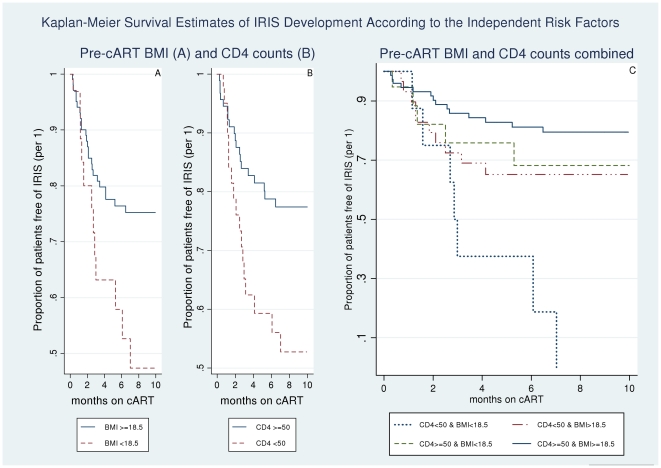
Kaplan Meier Survival estimates of IRIS development by the independent predictors identified. Kaplan Meier survival estimates of immune reconstitution inflammatory syndrome (IRIS) development according to the two independent predictors identified: pre-cART body mass index (A), pre-cART CD4 count (B), and the combination of both risk factors (C).

Kaplan–Meier estimates for the probability of remaining IRIS free by these 2 predictors are presented in Figure 1. Nineteen percent (14/74) of the patients with a pre-cART CD4 count ≥50 cells/µl and a BMI ≥18.5 had developed IRIS by 10 months on cART, whereas all 13 patients with a pre-cART CD4 count<50 cells/µl and a pre-cART BMI<18.5 either had developed IRIS (7/13, 54%) or had died (6/13, 46%) by month 7 (logrank test p = 0.01).

A sub-analysis was conducted to determine potential differences in IRIS predictors by mode of presentation and etiology. Both a baseline BMI <18.5 (HR 2.58, 95% CI 1.13–5.89, p = 0.024) and a pre-cART CD4 count <50 cells/µl (HR 2.37, 95% CI 1.08–5.21, p = 0.032) independently predicted the development of “unmasking” IRIS. In contrast, neither of these factors predicted “paradoxical” IRIS (HR 1.54, 95% CI 0.4–5.5, p = 0.67 for CD4 count <50 cells/µl, and HR 1.7, 95% CI 0.4–6.6, p = 0.43 for BMI<18.5). The only pre-cART variable associated with the development of “paradoxical” IRIS was a pre-cART CD4 percentage <7% (HR 8.6, 95% CI 1.1–68.8, p = 0.04). Pre-cART CD4 count <50 cells/µl, HIV RNA >5 log_10_ copies/ml and BMI <18.5 were independently associated with OI-IRIS, but only the BMI showed a trend for predicting MC-IRIS (Table 4).

**Table 4:** Multivariate analysis by Cox regression of predictors of IRIS development by etiology.

	OI-IRIS (n = 17)Multivariate HR (95% CI)	P value	MC-IRIS (n = 19)Multivariate HR (95% CI)	P value
**Body Mass Index**				
≥18.5	1		1	
<18.5	2.38 (0.91–6.2)	0.078	2.54 (0.9–7.2)	0.079
**CD4 counts**				
≥50 cells/µL	1		1	
<50 cells/µL	3.7 (1.4–9.6)	0.007	1.6 (0.6–4.36)	0.34
**HIV viral load**				
<5 log_10_ copies/ml	1		1	
≥5 log_10_ copies/ml	4.58 (1.05–20)	0.043	0.99 (0.39–2.5)	0.99

*OI-IRIS: IRIS associated with systemic OI; MC-IRIS: IRIS associated with muco-cutaneous manifestations.*

Neither a history of previous OI, the variation in CD4 or CD8 at 4 or 10 months, the proportion of naïve CD4 cells, activated CD4 or CD8 cells, nor the strength of the HIV virologic response were associated with IRIS development (Table 3).

## Discussion

This study describes the incidence and predictors of IRIS development in a rural sub-Saharan African setting, and suggests risk factors may differ by the mode of IRIS presentation and etiology.

The patients were representative of the AIDS population in SSA, with a predominance of young heterosexual women, ART naïve, and severely immunosupressed at cART initiation [18].

Twenty-six percent of the patients developed IRIS, consistent with previous studies [8], [9], [19], however IRIS incidence was higher than in a similar South African cohort (10.4%) [10]. Whereas this latter study was conducted in a tertiary hospital with thorough diagnostic evaluations performed prior to cART onset, our study was conducted in a district hospital with lower diagnostic facilities. This might have resulted in a better recognition and treatment of co-morbidities prior to cART initiation in the South African study and, as a consequence, a lower rate of IRIS development.

The median time to IRIS onset was 62 days and the majority of cases occurred within the first 3 months on cART. This concurs with previous studies [9], [10], and is thought to reflect a rapid restoration of functionally active cells in the first weeks after cART, leading to a poor regulation of the restored immune system and a paradoxical clinical worsening [20].

Almost half of our IRIS patients presented with systemic OI (OI-IRIS) and the remainder with muco-cutaneous manifestations (MC-IRIS). As expected [3], [10], [21], TB accounted for a high proportion of cases among OI-IRIS, whereas the proportion of KS-IRIS was surprisingly high [22], [23]. We have discussed this with more detail elsewhere [15]. Based on our results, it is plausible that KS-IRIS is more common than the literature reflects in certain areas of SSA, likely related to a high HHV-8 prevalence [24].

MC-IRIS has been previously described [25], and accounted for more than half of the IRIS episodes in our cohort, including herpes simplex infections, genital warts, herpes zoster, seborrheic dermatitis, folliculitis and tinea. There are few case reports of tinea associated with IRIS in western settings [7], [26]. However the high proportion observed in our cohort as well as in a previous South African study [27] suggests that dermatophytoses-IRIS might be common in areas with high endemicity of dermatophyte infections [28], [29].

Approximately 70% of the IRIS cases in our study were “unmasking” IRIS, which may be the most common form observed in SSA [10], [17]. This is likely due to both a higher burden of OI and to limited diagnostic facilities in rural Africa, and stresses the need for a careful evaluation of OI prior to cART initiation in these areas.

CART was continued in all IRIS cases and almost a third required anti-inflammatory treatment. The addition of anti-inflammatories was not associated with a different outcome, but the sample size was not powered to draw conclusions on IRIS treatment. A recent trial has demonstrated a beneficial effect of prednisone in the treatment of “paradoxical” TB-IRIS [30], and further similar studies should evaluate different therapeutic strategies for other pathogen-specific IRIS.

Two independent pre-cART IRIS predictors were identified, including a CD4 count <50 cells/µl and a BMI <18.5. Both predictors reflect advanced immunosupression, which is accompanied by a higher risk of OI, a residual inflammatory state, a persistent dysfunction of T-cells, and a disruption of homeostatic regulatory mechanisms [20], [31], [32]. Low BMI could also reflect unrecognized co-morbidities (such as disseminated TB, *Mycobacterium avium* complex disease, or cryptosporidiosis), and it could be useful to predict IRIS in rural African settings with low diagnostic facilities. Interestingly, the combination of both predictors was associated with a greater than 5-fold hazard of IRIS compared to the patients with both a CD4 count ≥50 cells/µl and BMI ≥18.5. These results suggest than initiation of cART at higher CD4 counts may result in a lower IRIS incidence, in addition to a known lower risk of disease progression [33], [34].

Our results also suggest that both the pre-cART CD4 count and BMI could be relevant predictors for OI-IRIS development, but not for MC-IRIS. They also appear to better predict “unmasking” than “paradoxical” IRIS, where the baseline CD4 percentage better identified patients at risk. However, these results must be interpreted with caution, due to the small number of events in each category.

IRIS is a heterogeneous condition and it is likely that both the immunopathogenic mechanisms and the predictors of its development are determined by the associated pathogen [20]. Interestingly, in a previous sub-analysis of our cohort, high baseline HIV viral load was identified as an independent predictor of KS-IRIS [15]. Further studies assessing predictors for pathogen-specific IRIS are needed.

Neither CD4 nor CD8 activation, as defined in this study, were found to be risk factors for the development of IRIS. A recent study has found that pre-cART expression of certain markers such as PD-1, a marker of T-cell exhaustion, may be associated with development of IRIS [35]. However, pre-cART CD38 and HLA-DR expression do not appear to predict IRIS development.

Three deaths were directly attributed to IRIS (8.3%), highlighting that, although most IRIS cases were mild, IRIS can be aggressive and hard to manage in resource-poor settings.

All IRIS studies are limited by the lack of confirmatory diagnostic tests and an internationally accepted IRIS definition. We used a strict definition and patients were identified prospectively, only considering previously reported etiologies of IRIS, and requiring the agreement of two clinicians to establish a final diagnosis. Other studies have used different definitions, and in order to compare studies, it is urgent to develop a consensus definition of IRIS and of pathogen-specific IRIS, such as the one existing for TB-IRIS [36].

Another limitation of our study is the use of a passive case-finding approach, possibly leading to an overestimation of the time to IRIS and to under-recognition of certain self-limited IRIS presentations. Nevertheless, 89% of the cases were diagnosed in non-scheduled visits, suggesting an active health-seeking behaviour in our patients, and the median time to IRIS after cART concurs with previous data.

In conclusion, with the ART scaling-up in rural SSA, awareness of IRIS is increasingly important. IRIS clinical spectrum varies according to the setting and it is thus, important to have case series and analysis of IRIS predictors from different areas. We have identified risk factors for IRIS development in a rural African setting which can easily be assessed at cART initiation. These predictors can aid in the selection of patients at a higher risk of IRIS for whom a closer monitoring is warranted. Furthermore, our findings stress the need for initiating cART at higher CD4 counts in SSA.
